# Cross-cultural adaptation and validation of the Chinese version of the PSS-QoL questionnaire

**DOI:** 10.1371/journal.pone.0313839

**Published:** 2024-12-05

**Authors:** Yu Zhou, Linli Zhuang, Xiaoqin He, Li Xu, Qi He, Xuemei Li, Yali Ye

**Affiliations:** Department of Rheumatology and Immunology, West China Hospital of Sichuan University/West China School of Nursing, Sichuan University, Chengdu, Sichuan, People’s Republic of China; King Abdulaziz University Faculty of Medicine, SAUDI ARABIA

## Abstract

**Background:**

The quality of life(QoL) of patients with primary Sjögren’s syndrome(PSS) is affected by a variety of symptoms, and it is important to comprehensively assess the factors affecting patients’ QoL.The PSS-QoL is a specific tool for the assessment of patients’ QoL in the PSS. The purpose of this study was to cross-culturally adapt the PSS-QoL for the Chinese language,to establish a QoL assessment tool for PSS patients in Chinese culture and to test the reliability and validity of the PSS-QoL.

**Methods:**

Our study period was from January 17, 2024 to June 15, 2024. The study was designed as a two-stage observational study. Double forward and backward translations of the PSS-QoL were performed for cultural adaptation to the Chinese language,and the specific steps included forward and backward translations, coordination, expert correspondence, small-sample surveys, and corrections to form the final draft of the Chinese version of the PSS-QoL. From January 29, 2024 to June 15, 2024,for the evaluation of psychometric properties, 135 patients with PSS completed the Chinese version of the PSS-QoL,Short Form-12(SF-12) and EULAR Sjögren’s Syndrome Patient Reported Index (ESSPRI).After 2 to 4 weeks,15 patients with PSS completed the Chinese version of the PSS-QoL for the second time.We used SPSS 26.0 software to statistically analyze the data, including item analysis and reliability and validity tests.

**Results:**

The Chinese version of the PSS-QoL consists of 25 questions and can be divided into two main categories: physical (discomfort and dryness) and psychosocial. The mean score on the Chinese version of the PSS-QoL was 40.46±15.00. Among the 135 patients with PSS,92.60% were female, the mean (±SD) age was 52.76±12.74 years, and the disease duration was 5 (1.5, 9) years. There was good differentiation between the individual items and the Chinese version of the PSS-QoL,as the decision value of all the items ranged from -3.223 to -12.234 (p < 0.05), the correlation coefficient between the individual items and the whole questionnaire ranged from 0.315 to 0.730 (p < 0.05), the Chinese version of the PSS-QoL had good reliability and validity,as Cronbach’s α = 0.886, the Spearman-Brown coefficient was 0.782,the reliability of the individual items was 0.89,and the values of the I-CVI and S-CVI/AVE were all 1, indicating good psychometric properties. The construct validity between the Chinese version of the PSS-QoL and the ESSPRI was excellent (p = 0.506 ~0.687), and that between the PSS-QoL and the SF-12 was good(p = -0.464 ~ -0.673).

**Conclusion:**

The Chinese version of the PSS-QoL possesses good reliability and validity, and all the indicies meet the metrics and satisfy the psychometrically acceptable range;therefore, it can be used as a reliable instrument for assessing the QoL of patients with PSS in China.

## Introduction

Sjögren’s syndrome(SS) is a complex chronic systemic autoimmune disease that is divided into primary Sjögren’s syndrome and secondary Sjögren’s syndrome and is more common in females, with a male to female ratio of approximately 1:9–14 [1]. SS mainly affects the lacrimal and salivary glands and is characterized by dry eyes and dry mouth. In addition, it may involve other systems, such as arthritis, cutaneous vasculitis, lymphoma, and lung diseases [2].The overall prevalence of SS has been reported to be approximately 0.007%, with a higher prevalence of approximately 0.043% in Europe and Asia [1],and the prevalence was 0.012% in Taiwan, China [3].Studies have shown [4] that the poor QoL of patients with PSS is mainly affected by a wide range of symptoms, such as dry mouth, dry eyes, pain, fatigue, dysphagia, taste dysfunction, itchy skin, sexual dysfunction, sleep disorders, anxiety, depression, and loneliness. Although the relevant symptoms do not directly jeopardize the lives of patients, long-term symptoms not only impose heavy physical, psychological and social burdens on patients but also seriously affect their QoL.

With the development of individualized medicine models and the promotion of patient-centered medical concepts, the status and role of patients in health decision-making are becoming increasingly prominent, and patient-reported outcomes (PROs) are increasingly emphasized. PROs refer to the symptoms of disease, health status or health-related quality of life reported entirely by the patient himself or herself, and PROs emphasize the patient’s subjective feelings, which compensates for the disadvantages of using objective indicators alone in clinical practice, which cannot accurately assess the degree of impact of the disease on the patient and the effectiveness of medical measures to treat the disease [5].Various PRO measurements have certain advantages and limitations. At present, PROMs such as the Short Form-36 (SF-36) [6], EuroQoL-5 dimension (EQ-5D) [7], Hospital Anxiety and Depression Scale (HADS) [6] are widely used to assess the QoL of patients with PSS. However, it has been found [8–10] that various symptoms, such as dryness, fatigue, and sleep disturbances, are important factors affecting the QoL of patients, yet the above general scales fail to reflect the symptom burden of patients. The ESSPRI [11] is a scale specifically designed to assess pain, fatigue, and dryness in patients with SS, which partially reflects the patient’s symptom burden. The Fatigue and Discomfort-Sicca Symptoms Inventory (short form) (PROFAD-SSI-SF) [12] is another PRO tool that can capture the impact of fatigue levels and dryness on patients’ daily lives; however, the problems are not limited to dryness, pain and fatigue, while the complaints secondary to these symptoms are important to patients and significantly affect physical, psychological and social life components of QoL, and problems such as digestive symptoms [13] and sleep disturbances [14]are important causes of the decline in quality of life in patients with PSS. Furthermore, symptoms such as dryness and pain may also cause some psychological problems, such as dry mouth, which may lead to difficulty speaking and swallowing, and patients may suffer from anxiety, depression, loneliness, embarrassment, and other psychological disturbances as a result, which may lead to a decrease in the QoL [15];however the ESSPRI and PROFDA-SSI-SF are unable to reflect these emotional burdens. In summary, none of the current general scales in China can fully reflect the physical, psychological and social problems of patients with PSS.

In 2018, Professor Lackner’s team [16] developed the first specific questionnaire for QoL in patients with PSS; this questionnaire has good reliability and validity, as the Cronbach’s coefficient is 0.892, and it not only comprehensively evaluates the symptom burden of patients but also reflects patients’ psychosocial problems. Currently, the feasibility and reliability of the PSS-QoL have been confirmed in the population of patients with PSS in Serbia [17], Turkey [18] and Romania [19]. Therefore, the aim of this study was to introduce a tool for evaluating and analyzing the QoL of patients with PSS in China through a scientific approach and to adapt and revise it to the Chinese cultural context so that it can meet the needs of healthcare professionals and patients in China regarding symptom burden, quality of life assessment and supportive help.

## Materials and methods

### Study design

The study involved adult participants who were inpatients and outpatients from the Department of Rheumatology and Immunology, West China Hospital, Sichuan University, China. Our study was divided into two phases. In the first phase, we translated the English version of the PSS-QoL into Chinese and adapted it cross-culturally, in the second phase of the study, a cross-sectional research design was employed and we tested the psychometric properties of the Chinese version of the PSS-QoL.

### Ethical statement

The study was approved by the institutional review board of the West China Hospital of Sichuan University(Approval No. of ethics committee: 2024 Annual Review No. 36), and written informed consent was obtained from each patient.

### Stage 1. Translation and cross-cultural adaptation

#### The PSS-QoL was translated into Chinese

This study was authorized by Professor Lackner, the original author of the PSS-QoL, and the original questionnaire was created based on a modified version of the Brislin back-translation model [20], with the following main steps: proper translation, synthesis and coordination, back-translation, expert review and finalization of the presurvey version by the subject team, presurvey and expert consultation, and the final formation of the Chinese version of the PSS-QoL. (1) Formal translation: The PSS-QoL was translated into Chinese by two people who were proficient in English, a nursing doctorate in mental health research and a nursing master’s degree in rheumatological and immune disease nursing. (2) Synthesis and coordination: The group coordinated with the translators to discuss and revise the differences in the original translation until a consensus was reached to form the Chinese version I. (3) The Chinese version I was back-translated into the English version I by two people who were proficient in both Chinese and English, one with a master’s degree in English language education, and one with a doctoral degree in English as a native language who had studied in China. (4) Expert review: Two nursing education experts familiar with the Chinese version of the scale were invited to compare the two back-translated English versions with the original PSS-QoL, and the group combined the experts’ modifications to form the English version II. (5) Finalization of the presurvey version by the research group: The group sent the English version II to Professor Lackner via email for review and approval. Professor Lackner highly approved the English version II and did not make any modifications, finally forming the Chinese version A of the presurvey PSS-QoL.

#### Cultural adaptation

Cultural adaptation was completed through a presurvey and expert consultation, and the investigators were members of the expert consultation group. (1) Presurvey: Twenty inpatients with PSS were selected for the presurvey via a convenience sampling method. After the questionnaire was completed, the patients were asked for their opinions, and those entries that were difficult for the patients to understand were modified to form Chinese version B of the PSS-QoL. (2) Expert Consultation: Five experts experienced in the diagnosis and treatment or nursing of patients with PSS were invited to comment on Chinese version B of the PSS-QoL, including one expert in the diagnosis and treatment of PSS, three experts in the nursing of PSS and one nursing professor engaged in the management of chronic diseases, with an average age of 45.40±9.07 years,working experience of 23.60±10.97 years, and academic qualifications of a bachelor’s degree or above, one with a full-senior title, one with a vice-senior title, and three with an intermediate title. The experts evaluated the PSS-QoL by combining theoretical knowledge and clinical experience and proposed modifications to the corresponding entries. The group integrated the opinions of patients and experts to form the final Chinese version of the PSS-QoL questionnaire.

### Stage 2. Psychometric investigation

#### Recruitment

Due to the low prevalence of PSS and in order to receive as many samples as possible, after discussion among the group we finally used a convenience sampling method, From January 29, 2024 to June 15, 2024, 135 patients with PSS in the outpatient and inpatient departments of the Department of Rheumatology and Immunology of a tertiary-level hospital in Chengdu city were selected as study subjects. The inclusion criteria for patients were as follows: were diagnosed with PSS based on the primary Sjögren’s syndrome diagnostic and treatment guidelines [21]formulated by the Chinese Medical Association, were aged >18 years, and voluntarily participated in this study. The exclusion criteria were as follows: critical condition could not cooperate with the investigation and the existence of communication barriers.

#### Sample size

According to standards for the respondent-to-item ratio [22], the sample size is usually 5~10 times the total number of items, the PSS-QoL questionnaire has a total of 25 items, and considering 10% invalid samples, the sample size is expected to be 138~275 cases, due to the low prevalence of PSS, time and energy limitations, a total of 138 questionnaires were distributed after discussion among the group, three invalid questionnaires were excluded (the three invalid questionnaires were not answered according to the questionnaire instructions), and finally 135 valid questionnaires were returned, with an effective recovery rate of 97.83%. The sample size for retest reliability was typically 10% of the total sample size, from which 15 patients were ultimately selected to be reassessed 2 ~ 4 weeks later for a retest reliability test.

#### Study instruments

In addition to the Chinese version of the PSS-QoL [16], the general information questionnaire, the ESSPRI and the SF-12 are also included.

The quality of life of questionnaire for patients with primary Sjögren’s syndrome [16]. The PSS-QoL was developed by Professor Lackner’s team in 2018 and contains 25 items on 2 dimensions—physiological (discomfort and dryness) and psychosocial,the content of which reflects the impact of various symptoms on patients’ QoL, such as somatic and psychosocial health. In the discomfort dimension, pain was scored using a numerical scale of 0 to 10, with 0 representing no pain and 10 representing unbearable pain, and the patient chose the appropriate scale according to the level of pain. Questions 2 to 6 were related to some uncomfortable symptoms, such as joint pain, digestive problems and sleep problems. Each of these questions had two options,‘yes’(scored 1) and ‘no’ (scored 0). Questions 7 to 11 consisted of entries related to dryness, such as dry mouth, dry eyes, dry skin and nasal dryness, of which question 11 was related to vaginal dryness, which was answered by women only. The psychosocial dimension consisted of 14 questions from question 12 to question 25. The answer to each question is given on a five-point Likert scale ranging from ‘never’ (scored 0), ‘seldom’(scored 1), ‘sometimes’(scored 2) and ‘often’ (scored 3) to ‘always’ (scored 4). Questions 15 and 20 were reverse scored from ‘never’ (scored 4) to ‘always’ (scored 0). The total scores on the PSS-QoL questionnaire ranged from 0 to 96 (women) and 0 to 92 (men, excluding vaginal dryness), with higher scores indicating poor QoL.The questionnaire took only 4~5 minutes to complete.

General information questionnaire, designed by the subject group, including sex, age, education level, marital status, occupation and disease duration.

The Short Form-12. The SF-12 has 12 items, including 8 dimensions of general health (GH, 1 entry), physical functioning (PF, 2 entries), physical functioning (RP, 2 entries), somatic pain (BP, 1 entry), vitality (VT, 1 entry), social functioning (SF, 1 entry), emotional functioning (RE, 2 entries), and mental health (MH, 2 entries). Scores for each dimension of the scale were converted into standardized scores with reference to the formula, with higher total scores indicating better QoL.

The EULAR Sjögren’s Syndrome Patient Reported Index. The ESSPRI [23] is used to assess pain, fatigue, and dryness in patients with SS and consists of three dimensions, each of which is scored using a numerical scale in which the patient scores the corresponding symptom based on his or her own subjective feelings, with a score ranging from 0 to 10 points for each dimension, with higher scores indicating a greater symptom burden for the patient.

#### Methods of data collection

The on-site questionnaire survey method was adopted, before conducting the survey, uniform training was provided to the investigators to enable them to understand the purpose of the survey, familiarize with the content of the questionnaire, avoid use specialized terminology and minimize communication bias. In the course of the investigation, One-to-one, face-to-face, line-by-line questioning was used for research subjects with literacy levels below primary school and older ages, and investigators interviewed patients in a neutral manner to ensure that the data collected was unbiased, while patients with literacy levels of junior high school and above completed the questionnaires on their own, which ensured the truthfulness of their answers, and the questionnaires were retrieved in a timely manner after they were completed on site.

#### Data analysis

SPSS 26.0 software was used to analyze the data, for descriptive statistical analysis, the count data are expressed as frequencies (percentages), We used the Kolmogorov-Smirnov test for normality of the measurement data, and the measurement data were expressed as mean ± standard deviations if they conformed to a normal distribution, and as median and quartile if they were not normally distributed, differences were considered to be statistically significant at P<0.05. The critical ratio method and item analysis method were used for the analysis of entries; the reliability test was evaluated by Cronbach’s coefficient, folded reliability and retest reliability; and the validity test was evaluated by expert consultation and the intragroup correlation coefficient for the content validity and validity scale validity of the Chinese version of the PSS-QoL, respectively. And physical discomfort, dryness and psychological aspects have been previously validated in the Serbian version [17] and the Romanian version [19], therefore, we did not conduct further factor analyses in this study.

## Results

### Results of cross-cultural adaptation

Most of the items in the PSS-QoL were in line with the Chinese language expression habits and cultural background, and the items were revised in five places during the cross-cultural adaptation stage. Some patients said they did not understand the meaning of ‘Dental problems’ in item 7 ‘Does your mouth feel dry?’, and after discussion by the group, it was decided to add the explanation as ‘Dental problems (such as blackened teeth, tooth loss, dental caries, dentures)’; the option ‘Gritty feeling’ in item 8 ‘Does your eyes feel dry?’ is a specialized term, which some patients expressed difficulties in understanding, and after discussion, it was supplemented with the explanation ‘Gritty feeling (the feeling of having sand in one’s eyes)’; Item 16 ‘I am too tired to fulfill obligations to my family and friends’ is a written expression, not a customary expression, revised to ‘I am too tired to fulfill obligations to my family and friends well (e.g., taking care of my family, visiting friends and relatives)’. For item 23, "Everyday activities such as driving, work, household and sports are a challenge", the experts suggested adding ‘doing farm work’, because some patients came from rural areas, and for farmers, doing farm work was part of their daily activities, so it was revised to ‘Everyday activities such as driving, work, sports, household and farming are a challenge for me’; for item 24, ‘Remedies such as eye drops, creams and physiotherapy impose a financial burden’, the experts suggested adding ‘oral medicines’, as patients with PSS often take oral medications to control their condition, was amended to read ‘Remedies such as eye drops, creams, physiotherapy and oral medications impose a financial burden’.

### General information on patients with PSS

The present study revealed that the patients with PSS were mainly female, with a male to female ratio of approximately 1:12.5, and approximately 90.37% were married, with an average age of 52.76±12.74 years, with the largest number of people aged 41~60 years, accounting for approximately 58.52% of the population, which is similar to the findings of another study [1]. The average duration of the disease was 5 (1.5, 9) years, and for the vast majority of the population, approximately 54.81% of patients had a secondary school education or higher. All demographic characteristics of the patients are listed in Table 1.

**Table 1:** Patient demographic characteristics (*n* = 135).

Categories	Project	Frequency (N)	Proportion (%)
**Sex**	Women	125	92.60
	Men	10	7.40
**Age (years)**	20~30	5	3.70
	31~40	18	13.33
	41~50	39	28.89
	51~60	40	29.63
	61~70	20	14.81
	71~80	13	9.63
**Education**	Junior high school and below	61	45.19
	High school and secondary school	29	21.48
	College and Bachelor’s Degree	42	31.11
	Postgraduate and above	3	2.22
**Marital status**	Single	5	3.70
	Married	122	90.37
	Divorced	3	2.22
	Widowed	5	3.70
**Employment status**	Employed	44	32.59
	Unemployed	56	41.48
	Retired	35	25.93

### Project analysis

#### Critical ratio

In this study, the total scores of 135 patients in the Chinese version of the PSS-QoL were arranged in ascending order from low to high, 37 patients (the first 27%) with a total score of less than 31 were classified as the low subgroup, 37 patients (the second 27%) with a total score of more than 49.28 were classified as the high subgroup, and the scores of the two groups were subjected to the t test of two independent samples. The results showed that the 25 items of the Chinese version of the PSS-QoL were significantly different between the high and low subgroups (P<0.001), suggesting that the individual items were well differentiated and that all the items were retained.

#### Correlation analysis

In this study, Pearson’s correlation analysis was used to test the homogeneity of the correlation between each item of the PSS-QoL and the total score. The results showed that each item of the Chinese version of the PSS-QoL was positively correlated with the total score, and except for item 6, the other correlation coefficients of the 24 items were all above 0.3 (0.315~0.730). The correlation coefficient of item 6, "I had sleeping problems", was 0.282 (<0.3), but P<0.001, and sleep disorders are very common in patients with PSS. As shown in one study [14], approximately 57.5% of PSS patients have sleep disorders. Compared with healthy controls, PSS patients have sleep problems such as a high number of awakenings and poor sleep quality, and patients with poor sleep quality are not only easily tired but also prone to some anxiety and depression, which seriously affects their QoL. Therefore, after discussion among the experts, it was recommended that the item be retained. The results are shown in Table 2.

**Table 2:** Correlation of individual items and total score.

Items	Correlation coefficient	
	*r*	*p*
**1**	0.538**	0.000**
**2**	0.399**	0.000**
**3**	0.427**	0.000**
**4**	0.315**	0.000**
**5**	0.462**	0.000**
**6**	0.282**	0.001**
**7**	0.538**	0.000**
**8**	0.548**	0.000**
**9**	0.552**	0.000**
**10**	0.386**	0.000**
**11**	0.351**	0.000**
**12**	0.574**	0.000**
**13**	0.412**	0.000**
**14**	0.697**	0.000**
**15**	0.352**	0.000**
**16**	0.701**	0.000**
**17**	0.640**	0.000**
**18**	0.564**	0.000**
**19**	0.519**	0.000**
**20**	0.336**	0.000**
**21**	0.716**	0.000**
**22**	0.708**	0.000**
**23**	0.695**	0.000**
**24**	0.629**	0.000**
**25**	0.730**	0.000**

**p<0.001.

### Validity

#### Content validity

A total of five experts rated the content validity of the Chinese version of the PSS-QoL in this study, with the same members of the expert group involved in cultural adjustment. The I-CVI and S-CVI/AVE values were calculated to be 1, and the experts recommended retaining all entries.

#### Validity of a criterion

In this study, the SF-12 and the ESSPRI were used as external calibrators for the Chinese version of the PSS-QoL, and the correlation coefficients between the dimensions of the SF-12 and the total scores of the Chinese version of the PSS-QoL were negatively correlated, with correlation coefficients ranging from -0.464 to -0.673 (P<0.001). The correlation coefficients of each dimension of the ESSPRI and the Chinese version of the PSS-QoL were all above 0.5, indicating that the Chinese version of the PSS-QoL was highly correlated with the ESSPRI, with a dryness dimension of 0.646, a pain dimension of 0.506, and a fatigue dimension of 0.687. The results are shown in Table 3.

**Table 3:** Correlations between the PSS-QoL and the SF-12 and ESSPRI.

	PSS-QoL Total score	Physical PSS-QoL	Discomfort PSS-QoL	Dryness PSS-QoL	Psychosocial PSS-QoL
**SF-12—GH**	-0.511**	-0.343**	-0.231**	-0.309**	-0.544**
**SF-12—PF**	-0.464**	-0.408**	-0.351**	-0.336**	-0.415**
**SF-12—RP**	-0.600**	-0.487**	-0.372**	-0.420**	-0.564**
**SF-12—RE**	-0.673**	-0.503**	-0.290**	-0.472**	-0.673**
**SF-12—BP**	-0.471**	-0.424**	-0.514**	-0.287**	-0.396**
**SF-12—VT**	-0.464**	-0.325**	n.s.	-0.317**	-0.490**
**SF-12—MH**	-0.488**	-0.284**	n.s.	-0.266**	-0.558**
**SF-12—SF**	-0.632**	-0.513**	-0.294**	-0.483**	-0.586**
**ESSPRI—dryness**	0.646**	0.542**	0.190*	0.559**	0.585**
**ESSPRI—pain**	0.506**	0.552**	0.839**	0.305**	0.353**

Abbreviations: *SF-12*, 12-Item Short-Form Health Survey; *GH*, general health; *PF*, physical function; *RP*, role-physical; *RE*, role-emotional; *BP*, bodily pain; *VT*, vitality; *MH*, mental health; *SF*, social functioning; *ESSPRI*, EULAR Sjögren’s Syndrome Patient Reported Index; *PSS-QoL*, Quality of Life in Primary Sjögren’s Syndrome.

** *P<*0.001

*P<0.05; n.s.–not significant.

### Reliability

In this study, Cronbach’s α was used to evaluate the internal consistency of each item of the Chinese version of the PSS-QoL, and the Cronbach’s α of the Chinese version of the PSS-QoL was 0.886, which indicated that the questionnaire had good internal consistency.

#### Split-half method

The Chinese version of the PSS-QoL was divided into two parts: the first part contained 13 questions, the second part contained 12 questions, and the correlation coefficients of the two groups were calculated as a reference for the folded reliability of the questionnaire. The results showed that the Cronbach’s alpha coefficient of the first part was 0.71, that of the second part was 0.881, and that of the Spearman-Brown coefficient was 0.782. Considering that the Spearman-Brown coefficient did not fall below 0.7 after the split-half method, the satisfactory reliability of the Chinese version of the PSS-QoL was further verified.

#### Retest reliability

In this study, a convenience sampling method was used to assess the retest reliability by distributing the same questionnaire after 2~4 weeks to 15 patients from 135 respondents. The test-retest reliability of the PSS-QoL was good, with a test-retest reliability of 0.89 and an ICC of 0.942 [95% CI: 0.885~0.977], which indicated the excellent reliability of the PSS-QoL. The mean values of the total and individual domain scores of the PSS-QoL at baseline and follow-up are given in Table 4.

**Table 4:** Mean values of the PSS-QoL dimensions and total scores at baseline and follow-up.

PSS-QoL	Baseline(mean±SD)	Follow-up(mean±SD)
**Score**	52.07±14.811	46.73±17.702
**Physical**	27.67±8.541	24.73±11.683
**Discomfort**	6.07±2.865	5.27±3.011
**Dryness**	18.20±6.581	16.53±8.400
**Psychosocial**	24.40±7.424	22.00±7.319

## Discussion

Based on a qualitative study [24], Professor Lackner’s team developed the first specific tool for the assessment of patients’ QoL in PSS, which explored the experiences and perspectives concerning QoL in patients with PSS. The final PSS-QoL questionnaire includes two dimensions, physical (discomfort and dryness) and psychosocial factors. This tool ensures that the content of the final PSS-QoL questionnaire is highly relevant to patients with PSS. After testing, the decision values of all items in the Chinese version of the PSS-QoL ranged from -3.223 to -12.234 (P<0.05), and the correlation coefficients of each item and the whole questionnaire ranged from 0.315 to 0.730 (P<0.05), which indicates that the Chinese version of the PSS-QoL has a good differentiation of each item. The reliability of the questionnaire reflects the reliability and stability, and commonly used reliability evaluation indices, including Cronbach’s α and retest reliability, and it is generally considered that a Cronbach’s α coefficient>0.8 and retest reliability>0.75 are good. The Chinese version of the PSS-QoL has a Cronbach’s α of 0.886, the reliability of the test was 0.89, and the ICC was 0.942, which are comparable to those of previously published versions, such as the English version of the PSS-QoL (Crohnbach’s α = 0.892, ICC = 0.958) [16], the Serbian version (Crohnbach’s α = 0.922, ICC = 0.981) [17], the Romanian version (Crohnbach’s α = 0.930, ICC = 0.971) [19] and the Turkish version (Crohnbach’s α = 0.955, ICC = 0.914) [18]. Validity reflects the validity and correctness of the questionnaire, and the commonly used validity indices are content validity and calibration validity. The values of the I-CVI and S-CVI/AVE were both 1, which met the requirements of reliability and content validity in the measurement indices, indicating that the Chinese version of the PSS-QoL has good validity. The tool can thus be used in clinical trials or practice as a validated outcome measure.

We used the SF-12 as the calibration scale for the Chinese version of the PSS-QoL, and the results showed that there was a good correlation between the SF-12 and the PSS-QoL. In other studies, the EQ-5D was chosen as the calibration scale for the PSS-QoL because of its shortness and wide application. Indeed, there was only a moderate correlation between the EQ-5D and the PSS-QoL in other versions [16–18],because the EQ-5D did not reflect the burden of sicca symptoms in PSS patients. In the Serbian version [17],they did not find a significant correlation between the EQ-5D-mobility, self-care, usual activity and the PSS-QoL even, while the EQ-5D-pain/discomfort correlated very strongly with the same PSS-QoL dimension, and the anxiety/depression of the EQ-5D had a moderate correlation with the psychosocial component of the PSS-QoL. After discussion in the group, we finally chose the SF-12 as the calibration scale for the PSS-QoL, which has more entries and dimensions than the EQ-5D and has greater sensitivity in QoL evaluation. We demonstrated that the correlation between the PSS-QoL and SF-12 was greater than that between the PSS-QoL and the EQ-5D.

Regarding the PSS-QoL and the ESSPRI, the results showed a strong correlation between the PSS-QoL and all of the ESSPRI dimensions, which is similar to the results of other versions [16–18]. In addition, the ESSPRI pain score and PSS-QoL discomfort score were strongly correlated, and there was also a strong correlation between the ESSPRI fatigue score and PSS-QoL psychosocial score.

This is the first specific patient-reported outcome measure (PROM) for QoL in the PSS. The PSS-QoL focuses on patients’ perspectives and aspects of QoL, which may differ from clinical outcomes or concerns. The Chinese version of the PSS-QoL reflects the important factors affecting the quality of life of PSS patients, and studies have shown [24] that the quality of life of PSS patients is mainly affected by their wide range of symptoms. The PSS-QoL includes the symptoms most commonly experienced by PSS patients, such as pain, dryness and fatigue. The physiological dimension of the PSS-QoL not only includes the assessment of the impact of pain, digestive symptoms, sleep disorders and dryness on patients’ daily life but also includes some complications caused by dryness, such as difficulty speaking, burning sensation in the mouth, and dental problems caused by dry mouth, which enables healthcare professionals to have a more in-depth understanding of the degree of dryness of the patient and the related problems caused by it, which specifically reflects the burden of the disease. Therefore, the PSS-QoL has greater specificity and greater validity than universal QoL assessment scales such as the SF-12 and Euro-QoL-5D. To date, PSS-specific PROMs mainly include the ESSPRI [11], the Profile of Fatigue and Discomfort (PROFAD) [12] and the Sicca Symptoms Inventory (SSI) [25], but none of these instruments have been designed to measure QoL. The ESSPRI mainly focuses on pain, fatigue and dryness. The PROFAD evaluates fatigue, and the SSI focuses on dryness. These tools only focus on some of the symptoms of PSS patients, ignoring the psychological and social problems caused by symptoms. For example, patients may have difficulty speaking because of dry mouth, and in the long run, they may easily refuse social activities because of difficulty speaking, which may lead to loneliness or even depression, while the psychosocial dimensions of the PSS-QoL aptly reflect patients’ emotional burden and social consequences of dryness. Therefore, the PSS-QoL enables a comprehensive assessment of the QoL level of patients with PSS in terms of their physical, psychological and social problems and identifies important influencing factors that can provide a basis for the subsequent development of interventions.

The strengths of this study include the large sample size. We collected a total of 135 questionnaires, and although the sample size of this study was calculated according to the smallest multiplier (5 times), our sample size was much larger than that of other versions, such as the English version(75 cases) [16], the Turkish version(79 cases) [18], the Romanian version(52 cases) [19], and the Serbian version(30 cases) [17]; moreover, this questionnaire was developed with a detailed understanding of the psychological measurement indicators, instructions for use, and the background and development process of the questionnaire, and the authorization of Professor Lackner was obtained to complete the preparation and introduction of the questionnaire. After the introduction of the questionnaire, the Chinese version of the PSS-QoL was developed using scientific and rigorous methods of Chineseisation and cultural adaptation. The content validity assessment experts in this study had rich experience in PSS diagnosis and care, with an average working experience of 23.60±10.97 years. In addition, the questionnaire investigators underwent unified training and were qualified to participate in the survey before they started to conduct the survey. Data entry was performed by a two-person team, so the Chinese version of the PSS-QoL was developed through the process of preparation and introduction, and the sinicization of the PSS-QoL to cultural adaptation, implementation and data entry were all rigorous and scientific.

There are some limitations to this study. Firstly, Convenience sampling method was used in this study, because it only includes individuals who visited the West China Hospital of Sichuan University, which could lead to selection bias. Second, another limitation is that our study included data from only one regional center, PSS-QoL Chinese version in different provinces of China is recommended. Thirdly, because of low prevalence of PSS, and absence of specific symptoms,which leads to delays in seeking medical help for patients, resulting in a smaller sample. In future studies, the sample size can be appropriately expanded, and a national multi-center study can be conducted to further test the applicability of the questionnaire in PSS patients to improve the use of the questionnaire.

## Conclusion

In summary, the Chinese version of the PSS-QoL has good reliability and validity, meets the recommended standard of the questionnaire, and can be used to assess the QoL of PSS patients in China.

## Supporting information

S1 File Data collection form. (PDF)

S2 File Study database. (XLS)
