# Supplementary material for: Cross-cultural adaptation and validation of the Chinese version of the PSS-QoL questionnaire
Source: PLoS One. 2024 Dec 5;19(12):e0313839. doi: 10.1371/journal.pone.0313839 (PMC11620550; doi:10.1371/journal.pone.0313839)
Supplement: S1 File — (PDF) [file pone.0313839.s001.pdf]

S1 File. Data collection form.

1. Age：（ ）
2. Gender：①Men ②Women
3. Employment status：①Employed ②Unemployed ③Retired
4. **Marital status：**①Single ②Married ③Divorced
5. **Education：**①Junior high school and below ②High school and secondary school ③College and Bachelor's Degree ④Postgraduate and above
6. How many years have you had primary Sjögren's syndrome? （ ）

PSS-QoL

These questions pertain to your complaints/symptoms within the *past four weeks.*

7.How severe is your pain?

**0**	1	2	3	4	5	6	7	8	9	**10**

No pain *Unbearable pain*

8.Do you have joint pain? ☐Yes ☐No

9.Do you have recurrent wandering pain? ☐Yes ☐No

Do you have digestive problems such as :

10.Constipation: ☐Yes ☐No

11.Stomach/abdominal pain: ☐Yes ☐No

12. Do you find it difficult to fall asleep/ do you have insomnia? ☐Yes ☐No

13.Does it feel like you have dryness of the mouth ? ☐Yes ☐No

If yes: please click on the following symptoms you have:

13.1. A Burning sensation in the mouth. ☐

13.2.Speaking difficulties. ☐

13.3.Dental issues.(such as blackened teeth, tooth loss, dental caries, dentures) ☐

13.4. Changes in sense of taste. ☐

13.5. Difficulty in eating dry foods. ☐

13.6. Sensation of excessive thirst at night. ☐

14.Does it feel like you have dryness of the eyes? ☐Yes ☐No

If yes: please click on the following symptoms you have:

14.1. Recurrent inflammation ☐

14.2. Pain ☐

14.3. Gritty feeling (the feeling of having sand in one's eyes) ☐

14.4. Crusty eyes/eyelids ☐

14.5. Deterioration of vision ☐

14.6. Lack of tears (inability to cry) ☐

14.7. Limited or impossible to perform daily life activities such as driving, reading and watching TV☐

15.Does it feel like you have dry skin? ☐Yes ☐No

If yes: please click on the following symptoms you have:

15.1. Skin reddening ☐

15.2. Tight, taut skin ☐

16.Does it feel like you have a dry nose ☐Yes ☐No

If yes: please click on the following symptoms you have:

16.1. Change in sense of smell ☐

16.2.Nosebleed ☐

The following questionnaire are to be answered by women only:

17.Do you have the sensation of dryness of the vagina? ☐Yes ☐No

If yes: please click on the following symptoms you have:

17.1. Any kind of vaginal pain ☐

17.2. Itching ☐

17.3. Pain with intercourse ☐

Please check to indicate how these statements apply to you

	Never	Seldom	Sometimes	Often	Always
I have a feeling that					
18. I feel as if I am the only person with above-mentioned symptoms	☐	☐	☐	☐	☐
19. My symptoms are not taken seriously	☐	☐	☐	☐	☐
20. My symptoms are unbearable	☐	☐	☐	☐	☐
21. My family and friends are supportive and understand my condition	☐	☐	☐	☐	☐
22. I feel too tired of fulfilling my obligations to family and friends	☐	☐	☐	☐	☐
23. l feel withdrawn from life	☐	☐	☐	☐	☐
24. I am concerned about the treatment side effects	☐	☐	☐	☐	☐
25. I am worried about the prognosis of the illness	☐	☐	☐	☐	☐
26. I am very content with my physical condition	☐	☐	☐	☐	☐
27. I am too tired to fulfill obligations to my family and friends well(e.g, taking care of my family, visiting friends and relatives)	☐	☐	☐	☐	☐
28. I easily get tired / I get tired easily	☐	☐	☐	☐	☐
29. 'Everyday activities such as driving, work, sports, household and farming are a challenge for me	☐	☐	☐	☐	☐
30. Remedies such as eye drops, creams, physiotherapy and oral medications impose a financial burden	☐	☐	☐	☐	☐
31. This illness has reduced my quality of life	☐	☐	☐	☐	☐

SF-12

1.In general, would you say your health is:

①Excellent ②Very Good ③Good ④Fair ⑤poor

2. Does your health now limit you in Moderate activities, such as moving a table, pushing a vacuum cleaner, bowling, or playing golf? If so, how much?

①Yes, limited a lot ②Yes, limited a little ③No, not limited at all

3. Does your health now limit you in Climbing several flights of stairs? If so. how much?

①Yes, limited a lot ②Yes, limited a little ③No, not limited at all

4. During the PAST FOUR WEEKS, how much of the time have you accomplished less tha n you would like with your work or other regular- daily activities as a result of your physical health?

①All of the time ②Most of the time ③Some of the time ④A little of the time ⑤None of the time

5. During the PAST FOUR WEEKS, how much of the time have you not done work or othe r activities as carefully as usual as a result of your physical health?

①All of the time ②Most of the time ③Some of the time ④A little of the time ⑤None of the time

6. During the PAST FOUR WEEKS, how much of the tune have you accomplished less tha n you would like with your work or other regular daily activities as a result of any emotional problems (such as feeling depressed or anxious)?

①All of the time ②Most of the time ③Some of the time ④A little of the time ⑤None of the time

7.During the PAST FOUR WEEKS, how much of the time have you not done work or other activities as carefully as usual as a result of any emotional problems (such as feeling depresse d or anxious)?

①All of the time ②Most of the time ③Some of the time ④A little of the time ⑤None of the time

8.During the PAST FOUR WEEKS, how much did pain interfere with your normal work (in clude both work outside the home and housework)?

①Not at all ②A little bit ③Moderately ③Quite a bit ⑤Extremely

9.How much of the time during the PAST FOUR WEEKS have you felt calm and peaceful?

①All of the time ②Most of the time ③Some of the time ④A little of the time ⑤None of the time

10.How much of the time during the PAST FOUR WEEKS did you have a lot of energy?

①All of the time ②Most of the time ③Some of the time ④A little of the time ⑤None of the time

11. How much of the time during the PAST FOUR WEEKS have you felt downhearted and depressed?

①All of the time ②Most of the time ③Some of the time ④A little of the time ⑤None of the time

12.During the PAST FOUR WEEKS, how much of the tune has your physical health or emot ional problems interfered with your social activities (like visiting with friends,relatives, etc.)?

①All of the time ②Most of the time ③Some of the time ④A little of the time ⑤None of the time

ESSPRI

1. *How severe is your pain?*

**0**	1	2	3	4	5	6	7	8	9	**10**

No pain *Unbearable pain*

2. *How severe is your fatigue?*

**0**	1	2	3	4	5	6	7	8	9	**10**

No fatigue *Unbearable fatigue*

3. *How severe is your dryness?*

**0**	1	2	3	4	5	6	7	8	9	**10**

No dryness *Unbearable dryness*
